# Neuroendocrine Abnormalities Following Traumatic Brain Injury: An Important Contributor to Neuropsychiatric Sequelae

**DOI:** 10.3389/fendo.2018.00176

**Published:** 2018-04-25

**Authors:** Amir M. Molaie, Jamie Maguire

**Affiliations:** ^1^Tufts University School of Medicine, Boston, MA, United States; ^2^Department of Neuroscience, Sackler School of Graduate Biomedical Sciences, Boston, MA, United States

**Keywords:** traumatic brain injury, hypopituitarism, anterior pituitary, neuropsychiatric symptoms, post-concussive syndrome

## Abstract

Neuropsychiatric symptoms following traumatic brain injury (TBI) are common and contribute negatively to TBI outcomes by reducing overall quality of life. The development of neurobehavioral sequelae, such as concentration deficits, depression, anxiety, fatigue, and loss of emotional well-being has historically been attributed to an ambiguous “post-concussive syndrome,” considered secondary to frank structural injury and axonal damage. However, recent research suggests that neuroendocrine dysfunction, specifically hypopituitarism, plays an important role in the etiology of these symptoms. This post-head trauma hypopituitarism (PHTH) has been shown in the past two decades to be a clinically prevalent phenomenon, and given the parallels between neuropsychiatric symptoms associated with non-TBI-induced hypopituitarism and those following TBI, it is now acknowledged that PHTH is likely a substantial contributor to these impairments. The current paper seeks to provide an overview of hypothesized pathophysiological mechanisms underlying neuroendocrine abnormalities after TBI, and to emphasize the significance of this phenomenon in the development of the neurobehavioral problems frequently seen after head trauma.

## Introduction

### Neuropsychiatric Sequelae of Traumatic Brain Injury (TBI)

Traumatic brain injury affects approximately 10 million people worldwide annually and 1.7 million in the U.S. alone (1–3). The most common TBIs occur secondary to motor vehicle trauma, and the incidence has been growing as low- and middle-income countries increase motor vehicle use, with a projected rise of 50% from 2002 to 2020 (4–6). Sports injuries are also frequent causes of TBI; in fact, almost 500,000 sports-related TBI emergency department visits were reported nationwide between 2006 and 2011 (7). In addition, more than 300,000 military service members have been diagnosed with combat-related TBI since 2000 (8).

A wide spectrum of neurologic sequelae of TBI has been described, ranging from cognitive problems to emotional and behavioral symptoms. Neuropsychiatric complaints in particular play a significant role in TBI morbidity (9). These include concentration problems, depression, anxiety, fatigue, and loss of emotional well-being, and occur in approximately 30% of brain injury patients (10–16). This constellation of neuropsychiatric symptoms has been recognized across all levels of TBI severity, and has historically been loosely classified as part of a general post-concussive syndrome, or PCS, that was thought to be due to brain injury *per se* (17). According to the World Health Organization’s International Classification of Diseases, PCS is defined as the presence of three or more of the following symptoms that must be present within the first month post-injury, including headache, dizziness, fatigue, irritability, insomnia, and concentration or memory difficulty (18). More long-term sequelae, such as major depression, alcohol abuse or dependence, panic disorder, or psychotic syndromes have also been noted after TBI, regardless of severity (19, 20).

The clinical relevance of these findings is becoming apparent as increasing numbers of patients with TBIs are surviving due to improved acute care, and therefore, are more commonly developing neuropsychiatric symptoms. Importantly, even as the lethality of weapons in the war zone has increased due to widespread use of improvised explosive devices, improvements in body armor have led to better survival rates of soldiers (21). Consequently, a higher proportion of wounded soldiers returns home with TBIs and subsequently develops various sequelae (22). The clear decrease in quality of life (QoL) in patients suffering from these symptoms has prompted a thorough investigation of the pathophysiology behind them, and has led to exciting new fronts regarding the possible mechanisms of these post-TBI complications. The current paper will highlight hypothesized pathophysiological mechanisms of one likely culprit of neurobehavioral symptoms: pituitary function abnormalities associated with head trauma.

### The Pituitary Gland

The pituitary sits in a pocket of bone called the sella turcica of the sphenoid bone and is covered by a layer of dura mater called the diaphragm sella. A small organ, approximately the size of a pea and normally weighing less than a gram, the pituitary gland consists of distinct sections: the pars distalis, pars tuberalis, and pars intermedia [the three of which compose the anterior pituitary (AP)], as well as the pars nervosa (the posterior pituitary). The pars distalis describes the bulk of the AP, while the tuberalis designates the sheath that joins the infundibular stalk. The pars intermedia, situated between the pars distalis and nervosa, is responsible for only a minor portion of pituitary function. The AP releases growth hormone (GH), adrenocorticotropic hormone (ACTH), gonadotropins, thyroid-stimulating hormone (TSH), and prolactin after being directed to do so by specific hypothalamic activating hormones that arrive to the AP *via* the hypophyseal-portal capillary network (with the exception of prolactin, which is primarily released through the removal of tonic inhibition by dopamine). The posterior pituitary, by contrast, secretes anti-diuretic hormone (ADH) and oxytocin after receiving neural input from the hypothalamic tract (23).

### Neuroendocrine Dysfunction Following TBI

There is increasing evidence that pituitary disruption is a common consequence of TBI, as well as a likely contributor to PCS-like symptoms. Posterior pituitary abnormalities, namely of ADH, such as in syndrome of inappropriate secretion of ADH or diabetes insipidus, are already well-recognized consequences of TBI, given their clinically apparent presentations of fluid balance dysregulation in the body. Clinicians have historically missed AP abnormalities following head injury, however, likely as a result of their more variable clinical presentations, as well as the subtlety of their symptoms, which can lead to underreporting by patients.

The first case of post-head trauma hypopituitarism (PHTH) was reported in 1918 (24), though the syndrome did not garner significant attention until the turn of this century. Since then, a large body of research has demonstrated the importance of PHTH (25–33), even from minor trauma (34), such as sport-related concussions (35–37) and blast-related mild TBIs in veterans (38–40). Kelly and colleagues were the first group to systematically evaluate the prevalence of hypopituitarism long-term after TBI (25). Examining 22 patients with mild, moderate, and severe TBIs at least 3 months out from a head injury (median of 26 months), the authors found that 36.4% of patients had subnormal responses in at least one hormonal axis. The most substantial impairment found was that of GH, with 18.2% of patients provoking an inadequate response to the insulin tolerance test (ITT). Regarding other hormones, one patient presented with corticotroph response impairment and one with gonadotropin deficiency.

Later, a notable large-scale study designed by Agha et al. examined 102 patients at least 6 months post-injury (median of 19 months) (27). The authors used more stringent measures for defining hypopituitarism by reporting patients as GH deficient only if they failed in both the glucagon stimulation test and the ITT, thereby confirming clinical deficiency. Despite the stricter guidelines, GH deficiency (GHD) was still found in 10.7% of patients and severe GHD was indicated in 8.8%, as defined by the arginine + GH releasing hormone (GHRH) test. Deficiency in the corticotropic axis was likewise defined using two provocative tests rendering a 12.7% prevalence of ACTH deficiency. Moreover, 11.8% had evidence of gonadotropin deficiency, though this was also significantly correlated with age.

More recently, Abadi and others conducted a prospective study of 75 patients with moderate TBI, assessing pituitary function 3 and 6 months post-injury (32). Overall, 48% of patients demonstrated at least one AP abnormality; the most prevalent was insulin-like growth factor 1 (IGF-1) insufficiency, found in 24% of patients after 3 months. Circulating levels of IGF-1, which is produced by the liver per GH stimulation, can be used to indicate anomalies in somatotroph function. It is important to mention that low IGF-1 concentration is reasonably specific for GHD; however, its diagnostic sensitivity is low and, therefore, normal or even elevated levels cannot exclude GHD (41). Gonadotropin and cortisol deficiencies occurred in 16 and 13.3% of patients, respectively, at the 3-month assessment. Notably, these percentages did decrease at the 6-month timepoint.

Overall, the most frequent abnormalities reported are GHD, ACTH insufficiency, and gonadotropin deficiency (29, 32, 42–44). Yet, taken together, results from prospective studies on both short- and long-term endocrinopathies are quite varied in terms of purported incidence, ranging generally from 10 to 58% (38, 45, 46). Therefore, the literature is still currently divided on the clinical pertinence of PHTH, and while some claim that it represents an under-diagnosed phenomenon, others argue it does not occur as commonly as proponents propose (47, 48). It has been suggested that because many studies on PHTH did not perform secondary confirmatory testing, the reported prevalence of pituitary deficiencies is inflated. Furthermore, it is important to acknowledge that using various tests, assays, and cutoffs has a significant effect on the purported deficiency rates (49, 50). For instance, Klose et al. measured a prevalence of GHD in as low as 1% of TBI patients depending on the guidelines used (49). In a very recent opinion article, Klose and Feldt-Rasmussen propose that the high percentages reported in the literature are secondary to short-term studies of favorably selected patient groups (48).

Despite disagreement on the exact prevalence of PHTH, most studies and reviews demonstrate that hormone disruptions do occur in some TBI-affected individuals, as outlined above. In fact, recent recommendations offered by Tan et al. propose that TBI patients should obtain pituitary screening at 3–6 months post-injury if they underwent prolonged hospital stays, or show symptoms consistent with pituitary dysfunction (44). Interested readers can refer to thorough reviews by Tanriverdi and colleagues (43) and Tan et al. (44), which summarize studies done to date on the prevalence of chronic PHTH, discuss the frequency of various deficiencies, and comment on the methods used in experiments.

Interestingly, in patients with chronic hypopituitary issues not secondary to head trauma, similar complaints of fatigue, depression, anxiety, and loss of emotional well-being also arise (51). GHD in particular is associated with diminished psychological health and decreased QoL in adults, and since about 30 years ago has been designated as its own specific clinical syndrome (52). Research has revealed significant decreases in energy and emotional lability, heightened sense of social isolation, greater difficulties with sexual relationships, decreased QoL, and greater psychological distress in untreated versus treated GHD (53–55). Furthermore, it has been repeatedly demonstrated that post-TBI GHD is similarly associated with impaired motivation, depression, and decreased QoL, with no convincing role of trauma severity (40, 56–58). Therefore, it comes as no surprise that these same symptoms after head trauma that were once regarded simply under the umbrella of post-concussive syndrome are being reconsidered in the context of PHTH and specifically post-TBI GHD. Moreover, many symptoms of post-traumatic stress disorder (PTSD) overlap with those of TBI, and questions have arisen whether neuroendocrine pathologies may contribute to PTSD in the setting of head trauma (59, 60). Although it is extremely complicated to parse out which symptoms attributed to PTSD may be specifically linked to brain injury as such, knowing what symptoms may be due to neuroendocrine abnormalities is being given special attention (61).

## Pathophysiology of TBI and PHTH

### The Typical Neuroendocrine Response of the Pituitary to Stress or Injury

In examining endocrinopathies after TBI, it is important to first consider the standard stress response to injury that affects cortisol and other endocrine hormones. In the acute phase of the response following trauma (accepted as within the first 24 h post-injury) and subacutely (several days after injury), a predictable neuroendocrine sequence occurs that is well-documented (62). There is a biphasic pattern of acute metabolic changes described as an “ebb” and a “flow.” First, a sympathoadrenal storm takes place in the first few hours post-injury which characterizes the “ebb” phase. This consists of a decrease in overall energy expenditure as the body and brain attempt to protect homeostasis by altering normal tissue perfusion. Next, a “flow” phase takes over, during which catabolic processes provide substrates for trauma repair (63). Overall, the acute and subacute periods following injury exhibit elevated AP function. GH is known to participate in widespread signaling that contributes to cellular recovery after trauma, and augmented ACTH release promotes hypercortisolism in an effort to metabolically support the body while healing takes place, protect against excessive immune responses, and recover hemodynamic status (64, 65).

Until recently, it was assumed that the cellular and metabolic events that take place during the acute and subacute phases continue throughout the course of long-term illness. However, findings of late suggest that the neuroendocrine response to injury changes in prolonged stress (66, 67). Van den Berghe et al. reported that during protracted critical illness, overall pituitary secretions are actually diminished: pulsatile release of ACTH, GH, TSH, and prolactin decrease (although notably hypercortisolemia continues *via* non-ACTH-mediated pathways) (65, 68). It remains an ongoing controversy whether these AP changes represent an adaptation to benefit survival or signify a dysfunction during protracted states of particularly critical illnesses, but current thought views chronic alterations as detrimental, and in fact something to therapeutically intervene in (65).

### The Neuroendocrine Response to TBI

The neuroendocrine and metabolic response to brain injury is thought to proceed similarly to extracerebral injury in the acute and subacute phases (69, 70). One must, therefore, consider based on Van den Berghe’s findings that chronic hypopituitarism observed after TBI may simply represent a response similar to that observed in protracted illness, rather than a unique pathologic phenomenon. However, certain points argue that chronic PHTH should instead be considered a distinct pathology. For example, the hypopituitarism Van der Berghe reported appeared to have a hypothalamic rather than pituitary origin, which differs from the likely sources of PHTH, as will be discussed below. Furthermore, most PHTH patients present with one or few AP abnormalities, whereas the changes seen in chronic critical illness take place on a broader level.

Recent experiments by Taylor and colleagues argue for TBI-specific mechanisms that modify the standard stress response to create PHTH. They showed that moderate TBI in adult male rats leads to altered glucocorticoid receptor and GABA receptor signaling which produces increased negative feedback control of the HPA axis, causing subsequent long-term dysregulation of the neuroendocrine stress response (71–73). This may be a significant key in explaining diminished ACTH seen after TBI. Notably, as will be discussed below, a similar sensitization to negative feedback of the HPA axis has been shown to occur in patients with PTSD, offering a clue to how psychiatric symptoms might manifest from neuroendocrine dysfunction (61).

### Mechanisms of Neuroendocrine Disruption After TBI

The specific pathophysiological changes that induce PHTH remain elusive. However, in addition to the reports by Taylor and colleagues documenting TBI-related damage to the stress response pathway (71–73), several other hypotheses have been offered that are backed by experimental data, including (i) vulnerability of the AP gland to damage and (ii) immune system involvement.

#### AP Damage

The pathophysiology of posttraumatic pituitary damage is complex, involving primary focal insults as well as secondary damage due to edema, hemorrhage, hypotension, and hypoxia (11). Swelling of the pituitary compresses the gland, as it sits within an inflexible bony compartment, roofed over with the diaphragm sella (74). Shearing forces from trauma can directly injure the pituitary gland itself or the infundibulum that connects it to the hypothalamus. Skull base and sella turcica fractures can produce focal damage and have been reported in cases of PHTH (75), although it should be acknowledged that most studies and reports of PHTH have been in patients without sellar fractures. Damage to the pituitary stalk could affect the pituitary hormone-producing chromophils in the pars tuberalis, namely gonadotroph, corticotroph, and thyrotroph cells, which produce gonadotropins, ACTH, and TSH, respectively (76, 77). The association of post-TBI hyperprolactinemia to PHTH is also thought to be secondary to pituitary stalk compression, leading to withdrawal of dopaminergic inhibitory control (78). In line with this, it is possible that structural damage to the infundibulum could also produce other post-TBI pituitary deficiencies by removing hypothalamic input. Direct damage to the hypothalamus, on the other hand, is not typically seen as a consistent or major pathological factor leading to chronic PHTH. However, a recent experiment using a model of intracranial hypertension did show increased apoptosis throughout the HPA axis, including the hypothalamus, pituitary, and hippocampus (79). Other pathologies related to the hypothalamus have been suggested: a recent study by Tümer et al. found heightened expression of oxidative stress mediators in the hypothalamus after blast-induced trauma in rats (80). Post-TBI autoimmunity directed at the hypothalamus has also been discovered (81) and will be explored shortly.

Ischemic injury is hypothesized to play a considerable role in PHTH pathology given the vascular vulnerability of the AP gland. The concept of AP sensitivity to ischemia was proposed many years ago (82) and has been supported since by anatomical, clinical, and autopsy evidence. Structurally, the anterior lobe of the pituitary derives its blood supply primarily from hypophyseal-portal circulation; namely, from the long portal vessels, which branch from the superior hypophyseal artery after it forms a capillary plexus in the median eminence. Running along the infundibulum to the AP, these vessels are predisposed to damage from mechanical forces, hypertension, hypotension, and edema (83, 84).

Regions of the AP, either located peripherally (and, therefore, distal to the main blood supply) or in areas not subject to sufficient collateral flow seem to be the most affected in PHTH. The long portal veins are generally recognized to supply 70–90% of the anterior lobe parenchyma, predominantly in the anterior central regions. By contrast, the short portal vessels, which branch off the inferior hypophyseal artery, supply the posterior pituitary as well as 10–30% of the adjacent AP posteriorly (83, 85). Somatotrophs, which produce GH and are the most abundant chromophil cells in the pituitary, tend to localize more in the lateral and peripheral regions (although they also scatter medially), which might make them particularly sensitive to ischemia due to their downstream position relative to the long portal vessels (86, 87). In cases of Sheehan’s syndrome, wherein postpartum hemorrhage causes ischemic necrosis of the pituitary, GH deficiency is also one of the earliest hormones lost (88).

After trauma, a small posterior area of the AP and the outermost layer immediately deep to the capsule tend to survive, likely secondary to collateral from the short portal vessels and a variable amount of extraportal end-arterial circulation, respectively (82, 83). Corticotropic cells, on the other hand, are clustered centrally in the AP and may not have such dual supply, and can, therefore, be susceptible to necrosis in cases of ischemic damage from TBI (77). Corticotrophs can also be found in the lateral wings of the AP, in the pars intermedia, and in the pars tuberalis. Gonadotrophs lie diffusely throughout the pars distalis and constitute the majority of the pars tuberalis (77, 87).

Some authors emphasize an anterolateral distribution of the vulnerable long portal vessels and designate an anteromedial distribution of the short portal vessels into the AP; this might explain the frequent damage to the lateral somatotrophs, as well as the infrequency of TSH deficiency, given that thyrotrophs cluster medially (11, 23). However, this distinction of lateral versus medial distribution is not firmly established in the field.

Many autopsy case reports also support the significance of pituitary vascular vulnerability in TBI and PHTH. Studies decades ago by Ceballos (89) and Kornblum and Fisher (90) of over 200 TBI subjects revealed AP necrosis in about 22% of patients. More recently, it was found by Benvenga et al. in a review of cases that approximately 1/3 of TBI fatalities demonstrated pituitary gland necrosis (91). Another recent histological study revealed acute adenohypophyseal infarcts in 43% of specimens (92).

Part of the difficulty of supporting this hypothesis in the past was the lack of systematic imaging studies that correlated PHTH with any focal pituitary pathological abnormalities. In their aforementioned review published in 2000, Benvenga and colleagues noted CT or MRI lesions of the pituitary or hypothalamus in all but 6.6% of PHTH patients (91). Since then, focused imaging studies have confirmed that pathologic vascular changes of the pituitary take place both in the acute and chronic phase after brain injury (74, 93). Maiya et al. found MRI irregularities consistent with edema, hemorrhage, or infarction in 30% of TBI patients (74). Furthermore, when directly assessing the relationship between PHTH and MRI findings, Schneider and colleagues observed a higher frequency of chronic pituitary imaging abnormalities in TBI patients with hypopituitarism compared to TBI patients without hypopituitarism (93). They described a pattern of pituitary volume reduction as well as signal inhomogeneities interpreted to represent hemorrhage, necrosis, or fibrosis. The loss of pituitary volume seen in the sella can be attributed to the combined effects of increased intracranial pressure and pituitary necrosis (94). Recently, a prospective study by Zheng et al. using MRI with diffusion-weighted imaging showed that compared to controls, TBI patients displayed decreased pituitary tissue water diffusivity, recognized as a marker for microstructural damage, such as white matter lesions (95). Further, they found that the TBI patients who demonstrated hypopituitarism exhibited lower values for water diffusivity than those without hypopituitarism.

Several studies in animal models of closed head injury have also confirmed pituitary damage with neuroendocrine dysfunction secondary to TBI. For example, Greco et al. demonstrated GH/IGF-1 axis disruptions and pituitary vascular damage in rats after repeated TBIs (96). Damage in the HPA axis upstream of the pituitary gland has also been found; for instance, neuronal apoptosis in the hippocampi of rats after head trauma was shown to be associated with decreased levels of IGF-1 (97). Furthermore, an intriguing mechanism established of late is the disruption of tanycyte function after cortical contusion injury (CCI) in mice (98). Tanycytes are specialized glia lining the third ventricle that mediate the crossing of blood-borne substances into the brain (99). The authors conclude that rupture of the tanycyte barrier likely contributed to the diminished GH levels seen in mice that underwent CCI.

#### Immune System Involvement

Several other hypotheses regarding the pathology of PHTH development have also been proposed and have empirical support. One such example relates to genetic differences in PHTH predisposition, namely through polymorphisms in apolipoprotein E (APO E) (38). APO E is a lipoprotein manufactured in the central nervous system, and is produced in greater quantities following injury. Studies have shown APO E inhibits the neuroinflammatory cascade after injury, with the APO E3 isoform doing so with greater effectiveness than APO E4 (100). In a study of 93 TBI patients, those with APO E3/E3 genotypes were significantly less likely to suffer from PHTH (101). Therefore, one might speculate that secondary insults in the form of neuroinflammation and cytokines may be important in pituitary pathology after TBI. Animal studies have also suggested a role of inflammation in PHTH. Kasturi and colleagues demonstrated increased expression of inflammatory mediators, such as interleukin IL-1β and glial fibrillary acidic protein (a marker of astrogliosis) in the cerebral cortex, hypothalamus, and AP of rats after CCI (102).

Another characteristic of inflammation also warrants attention: autoimmunity, and particularly the role of anti-hypothalamic antibodies (AHAs) and antipituitary antibodies (APAs) (81). The importance of autoimmunity in PHTH was first proposed in 2008 by Tanriverdi and colleagues after detecting APAs in 44.8% of TBI patients 3 years post-injury (103). No APAs were found in controls. They discovered that APA-positive individuals were at significantly higher risk for PHTH, and high APA titers correlated with lower GH response to stimulation by GHRH + GHRP-6 (GH releasing peptide). A subsequent 5-year prospective study of 25 of the 29 patients in this same TBI group revealed that 60% of patients had AHAs and 48% had APAs, and of those proportions, individuals who tested strongly positive for these antibodies remained more likely to have PHTH (104). This pathophysiological finding was also tested in individuals who sustained chronic/repetitive mild TBIs. Antibodies to the hypothalamus or pituitary were present in 21.3 and 22.9% of boxers, respectively, but again in no controls (105). AHA positivity was significantly associated with hypopituitarism; however, APA positivity in this instance was not. Altogether, these results lend credence to the importance of neuroinflammation in the pathogenesis of PHTH.

### Neural Circuitry Abnormalities and Post-TBI Neurobehavioral Symptoms

While PHTH is likely an important contributor to neuropsychiatric symptoms of TBI, other pathophysiological causes have also been hypothesized to play a role. Neuronal axons have been shown to be particularly susceptible to damage from TBI (106). In fact, diffuse axonal injury (DAI) due to inertial forces of rapid head acceleration/deceleration or rotation encompass arguably the most common and important pathologies, even after mild TBI (78, 107). Cytoskeletal breakdown and disruptions in axonal transport cause axonal swelling and disconnection from targets, leading to neuronal degeneration (108).

Until recently, assessing DAI *in vivo* has been a challenge, as conventional CT and MRI scanning were not able to detect such microscopic and pervasive changes. However, advances in the form of diffusion tensor imaging have furnished the potential to detect acute axonal changes indicative of DAI by measuring anisotropic movement of water molecules, and thereby characterizing white matter microstructural changes (109). In addition, resting-state functional MRI now affords researchers the ability to study the connectivity component of neural networks through measurement of the blood oxygenation level-dependent signal (110). These developments in neuroimaging have led to the understanding that structural compromise of neurons in DAI likely contributes significantly to functional disruptions of cognition by interfering with proper connections within certain neural circuits. The default mode network (DMN), for instance, is a functional circuit that describes the thoroughly investigated “resting state network.” The DMN is active during passive mental activities when the brain is not partaking in any specific goal-directed tasks requiring attention (111). Numerous studies have found decreased connectivity in this circuit following TBI, either from damage to important relay points (i.e., nodes) within the network or due to axonal injury between nodes (110). In addition, further abnormalities in the DMN after TBI were demonstrated specifically during attention-requiring tasks. Bonnelle et al. showed that moderate to severe TBI patients demonstrated abnormalities in DMN deactivation during a sustained attention task that were directly predicted by the magnitude of white matter damage within Salience Network tracts (a circuit which generally becomes active when engaging in tasks that solicit attention) (112). This is significant, as it was previously shown that a similar failure to inhibit the DMN correlates directly with lapses in attention during concentration-requiring tasks, a common sequela of TBI (113).

Given the circuitry changes seen in patients with TBI-induced concentration deficits, one might infer that interference with neural circuits is likely a significant contributor in provoking other neuropsychiatric symptoms as well. Consistent with this hypothesis, Zhou and others found that mild TBI patients demonstrated particular DMN dysfunction—specifically regarding connectivity changes around the medial prefrontal cortex (MPFC)—that were related to patient reports of depression, anxiety, and other PCS symptoms (114). However, the authors note that these DMN abnormalities are different from those seen in clinical anxiety disorder and depression. But they suggest that over the long term, the MPFC alterations seen after TBI could lead to similar persistent psychological problems.

Damage to certain frontal-subcortical circuits, which play important roles in cognition and social considerations, have also been predicted to cause disruptions in behavior after injury to pertinent nodal regions (115, 116). These circuits include (1) a network arising in the dorsolateral prefrontal cortex regulating executive functions (i.e., decision making, problem solving, working memory), (2) a network based in the orbitofrontal cortex, which modulates intuitive social reactions and comportment, and (3) a third originating in the anterior cingulate cortex that modulates motivation-related behavior. Animal studies have also supported the pertinence of prefrontal cortex remodeling in the pathogenesis of TBI (117). Overall, disturbances in functional neural circuits are being investigated thoroughly for answers regarding cognitive and neurobehavioral abnormalities after TBI, and may in fact overlap with disturbances in pituitary function. For instance, upstream circuits regulating neuroendocrine pathways, such as those traversing medial temporal structures like the hippocampus or amygdala—which are commonly injured in TBI—may result in dysfunction of the HPA axis due to impaired regulation (116).

Damage to similar brain areas as discussed here has also been implicated in the pathogenesis of PTSD (118). The pathophysiological basis of PTSD, however, is incredibly diverse and quite complex.

## The Mysterious Case of PTSD

While PTSD is not a frank sequela of TBI, PTSD and TBI are often comorbid and share many overlapping symptoms, such as fatigue, irritability, poor sleep, and concentration deficits (119, 120). The fact that they often coexist brings up the question of whether PTSD and TBI share a common pathological mechanism. At present, PTSD is recognized as a phenomenon related to a psychological insult bringing about structural or cellular changes, as brain injury is clearly not required for PTSD development. Therefore, even in patients with PTSD and TBI, physiologic damage from trauma is not seen as the inciting factor leading to the reorganization of brain activity patterns. Rather, the memory of the event and the associated psychic trauma is considered the etiology. It has even been proposed that amnesia associated with TBI might actually protect against PTSD, with a prospective study finding higher rates of PTSD among TBI patients who remembered the traumatic incident (121). Pharmacologic studies corroborate this notion: drug therapies aimed at inhibiting consolidation of emotionally disturbing memories prevent their subsequent recall (122, 123). In PTSD, propanolol given in conjunction with traumatic memory reactivation, or “re-consolidation,” has shown promise in decreasing symptoms (124, 125). However, the effect of TBI on the development of PTSD is still in dispute. It has been postulated that implicit processing of traumatic experiences takes place in spite of amnesia for the event, and that TBI does not reduce the frequency of PTSD (126). A military survey of soldiers returning from Iraq reported 44% of troops who had sustained mild TBIs with loss of consciousness screened positive for PTSD as compared to 16% who sustained only bodily injury (127). Additionally, a very recent study of veterans returning from dangerous posts in Iraq or Afghanistan found significant main effects of mild TBI on self-report questionnaires measuring PTSD symptoms (60). From an experimental angle, an animal study by Elder and others found that blast exposure causing mild TBI in anesthetized mice provoked PTSD-related traits, such as increased startle response (i.e., anxiety), enhanced contextual fear response, altered response in a predator scent assay, and increased expression of a protein involved in the fear response, suggesting that frank structural injury from the blast may have been the cause of the symptoms rather than memory of the event (120).

Currently, one of the predominant theories of PTSD comes from a neuroanatomical perspective that highlights the role of hyper-responsiveness of the amygdala to stimuli secondary to ineffective “top-down” processing by the prefrontal cortex (78). More recent theories have argued that disturbances between certain functional brain circuits, such as between the salience and DMNs, are partly to blame (128). Interestingly, these same neural circuitry abnormalities are seen in both TBI and PTSD (78). Overall, however, the vast heterogeneity of pathophysiology associated with PTSD poses a major challenge in making comparisons to TBI.

It is unclear to what degree PHTH might, if at all, contribute to the development of PTSD. The potential role of cortisol in PTSD was initially investigated intensively due to its significance in the stress pathway. It was assumed that PTSD patients would display elevated levels given the function of cortisol in the stress response. But numerous reports of PTSD patients with abnormally low corticosteroids challenged this assumption (61). Mason et al. were the first to demonstrate low cortisol levels in PTSD patients when comparing urinary free-cortisol levels between different psychiatric patient populations in the hospital (129). Many other studies followed; a notable study was performed by Resnick and colleagues, who studied cortisol levels within 51 h in victims of rape (130). They found that individuals with a history of previous assault displayed lower cortisol levels after a successive rape and had increased probability of developing PTSD. Related studies investigated the possible mechanisms behind this and have shown that PTSD patients have heightened sensitivity to glucocorticoid negative feedback and augmented negative feedback inhibition of the HPA axis as a whole (61). Recall, Taylor and colleagues proposed similar abnormalities in TBI (71–73). Additional research has given further credence to the notion that diminished cortisol response to stress/trauma may play a role in PTSD, but altogether the literature is substantially inconsistent. An abundance of studies have been conducted that yielded mixed results, finding decreased, increased, or unchanged basal levels of cortisol in PTSD patients and PTSD animal models, likely due to differences in the timing and type of trauma incurred (psychosocial, physical, acute, and chronic) (59). An excellent review by Zoladz and Diamond (61) delves into the conflicting literature regarding the neuroendocrine response and PTSD development. However, if low cortisol does correlate to likelihood of PTSD, one could imagine a role of PHTH in exacerbating or predisposing a person to developing this disorder. This is nonetheless speculative at this point. And yet, one aforementioned study of veterans did report intriguing results: using self-report questionnaires to investigate the relationship between TBI, overall hypopituitarism, and PTSD, the authors revealed a significant main effect of TBI-induced hypopituitarism on questionnaires assessing PTSD symptoms (60).

## Conclusion

Neuropsychiatric symptoms after TBI are common and impair QoL of survivors. Hypotheses concerning the pathological roots of these sequelae have pointed to a number of causes, of which PHTH, and particularly post-TBI GHD, is likely particularly important. Through pathophysiological pathways such as ischemic injury to the pituitary and immune-related mechanisms, head trauma frequently produces disruptions in AP function. As more prospective studies are done to ascertain the true prevalence of PHTH, the true contribution of these pathophysiological mechanisms to the development of neuropsychiatric symptoms will be elucidated. Future efforts should focus on further characterizing and identifying pituitary damage after TBI using prospective analyses with precise neuroimaging. In addition, it is imperative to employ more animal models of head injury to uncover the mechanisms by which PHTH occurs. Last, a goal toward teasing out associations between TBI and PTSD should be set, with a specific emphasis on possible neuroendocrine-mediated contributions.

## Author Contributions

AM and JM conceived and wrote the manuscript.

## Conflict of Interest Statement

The authors declare that the research was conducted in the absence of any commercial or financial relationships that could be construed as a potential conflict of interest. The reviewer BN declared a shared affiliation, with no collaboration, with the author JM to the handling Editor.
